# *In Vivo* Electroporation Mediated Gene Delivery to the Beating Heart

**DOI:** 10.1371/journal.pone.0014467

**Published:** 2010-12-30

**Authors:** Erick L. Ayuni, Amiq Gazdhar, Marie Noelle Giraud, Alexander Kadner, Mathias Gugger, Marco Cecchini, Thierry Caus, Thierry P. Carrel, Ralph A. Schmid, Hendrik T. Tevaearai

**Affiliations:** 1 Department of Cardiovascular Surgery, University Hospital of Berne, Berne, Switzerland; 2 Division of General Thoracic Surgery, University Hospital of Berne, Berne, Switzerland; 3 Department of Pathology, University of Berne, Berne, Switzerland; 4 Department of Urology, University Hospital of Berne, Berne, Switzerland; Leiden University Medical Center, Netherlands

## Abstract

Gene therapy may represent a promising alternative strategy for cardiac muscle regeneration. *In vivo* electroporation, a physical method of gene transfer, has recently evolved as an efficient method for gene transfer. In the current study, we investigated the efficiency and safety of a protocol involving *in vivo* electroporation for gene transfer to the beating heart. Adult male rats were anesthetised and the heart exposed through a left thoracotomy. Naked plasmid DNA was injected retrograde into the transiently occluded coronary sinus before the electric pulses were applied. Animals were sacrificed at specific time points and gene expression was detected. Results were compared to the group of animals where no electric pulses were applied. No post-procedure arrhythmia was observed. Left ventricular function was temporarily altered only in the group were high pulses were applied; CK-MB (Creatine kinase) and TNT (Troponin T) were also altered only in this group. Histology showed no signs of toxicity. Gene expression was highest at day one. Our results provide evidence that *in vivo* electroporation with an optimized protocol is a safe and effective tool for nonviral gene delivery to the beating heart. This method may be promising for clinical settings especially for perioperative gene delivery.

## Introduction

Coronary artery disease continues to be a major cause of morbidity and mortality worldwide. Currently, the medical treatment and/or myocardial revascularization procedure, either by percutaneous angioplasty or by coronary bypass surgery (CABG) remains the standard therapy [1]. Regeneration of the infracted myocardium, however, is the major challenge and its lack remains the predominant cause of death. New therapeutic modalities which may yield better and consistent results have to be evaluated. Gene therapy, the transfer of nucleic acids to achieve therapeutic benefits is one promising approach. However, progress towards effective human gene therapy in cardiovascular diseases has been hindered by a number of problems including vector toxicity, poor targeting of diseased tissues, and host immune and inflammatory activity [2], [3], [4]. Hence, safe and reproducible methods have to be established. Electroporation a physical method of gene transfer is one such promising technique.

It is based on the application of strong electric pulses for a very short duration to enhance transfer of macromolecules like DNA and proteins through cell membranes. Even though a number of hypothesis regarding the cell membrane permeabilization have been suggested [5], [6], the exact mechanism remains unclear. Nevertheless, electroporation has been shown to be one of the most efficient gene transfer strategies in vivo [7], [8] and was tested in a broad range of target tissues and organs [9], [10], [11], [12], [13], [14]. *Ex vivo* electroporation to the heart in experimental models has also been reported [15], [16] and showed a 100 to 1000 folds increase in transgene expression as compared to direct DNA injection. Consequently, the use of *in vivo* electroporation mediated transfer may represent a promising method to overcome the common limitations of gene therapy protocols using other types of vectors. Regarding cardiac gene delivery more specifically, direct injection into the cardiac muscle is the most common approach. Gene therapy protocols also involve antegrade coronary injection after aortic cross clamping, however, both approaches do not result in global vector distribution in the heart. Furthermore, the antegrade approach is also limited by heterogeneous and inefficient distribution in the presence of coronary stenosis or occlusion. To overcome this problem we present a retrograde approach by injection via the coronary veins after transient occlusion of the coronary sinus, to study the efficiency and the effect of *in vivo* electroporation mediated gene transfer to the beating heart.

## Materials and Methods

### Plasmid

The plasmids pCiKlux expresses firefly luciferase from the CMV immediate early promoter/enhancer as described before [13], [17]. EGFP plasmid was from Add gene (USA). The endotoxin-free plasmids were produced in large scale at Plasmid Factory GmbH & Co (Bielefeld, Germany). For electroporation, plasmids were suspended in endotoxin-free water.

### Animals

Inbreed male Wistar rats (200–220 g) (Janvier, France) were used and maintained on rodent feed and water in a air and temperature controlled room. The experiments were performed in compliance with the standards of the European convention of animal care. The study protocols permission numbers 32/03 and 21/06, were approved by the University of Berne Animal Studies Committee.

### Gene Transfer technique

Adult male rats were anesthetized by inhalation of 4% isoflurane in a glass chamber before being intubated and ventilated via a 14GA catheter (Insyte, Madrid, Spain) with FIO2 = 1.0 and 1.5% isoflurane to maintain anesthesia, a breathing frequency of 100/min, and a tidal volume of 10 ml/kg body weight with a rodent ventilator (model 683 Harvard Apparatus, South Natick MA).

**Figure 1:**
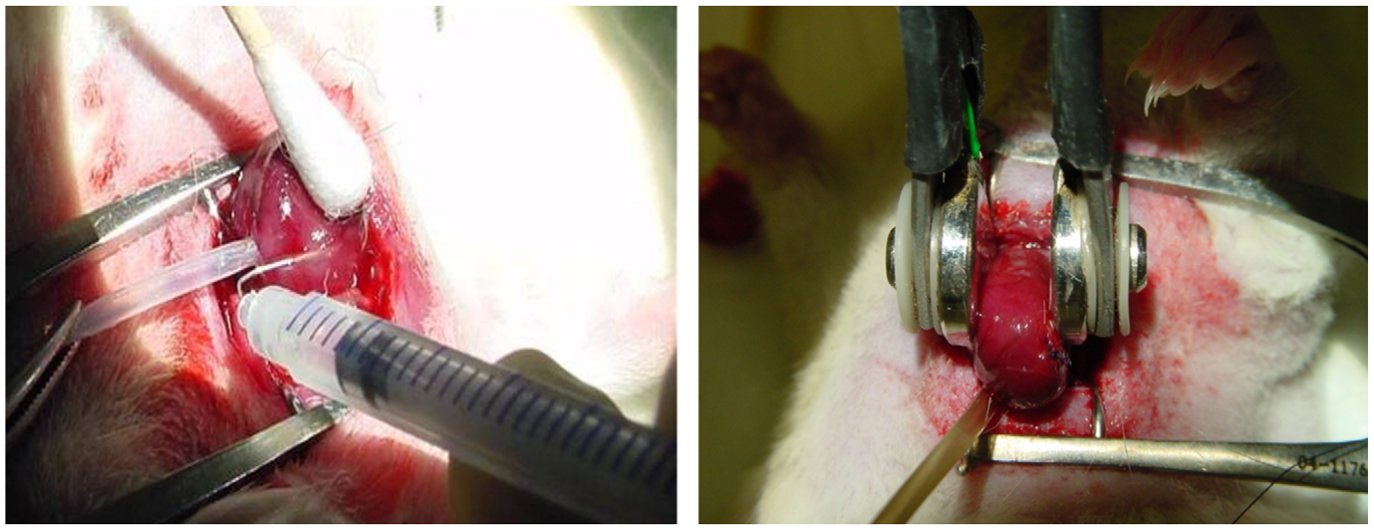
The *in vivo* electroporation mediated gene transfer to the heart is a two step procedure. (a) First, the coronary sinus is occluded with a 6-0 prolene tourniquet and the plasmid is injected into the coronary vein. (b) The heart is then positioned between the plate electrodes and the electric field is applied.

A left thoracotomy in the 4th intercostal space was performed and the heart was immobilized, the apex was lifted using an apical 7-0 prolene suture (Ethicon) and a 6-0 tourniquet was placed around the distal coronary vein to allow temporary coronary sinus occlusion (Figure 1a), immediately followed by the retrograde injection of plasmid solution in a volume of 150 µl (plasmid concentration 1 mg/ml). Animals in all groups below were injected with equal volume of plasmid. Plate electrodes (2 cm×1 cm) were placed on each side of the heart followed by the application of 8 pulses of 20 ms and 200 V/cm,1 Hz using the pulse generator (Inovio, San Diego) (Figure 1b). The distance between the two plates of electrode was 1.2 mm. The tourniquet was then immediately released. After the procedure, the heart was observed for 2 minutes before a small chest drain was inserted into the left hemithorax and the thoracotomy was closed with four layers of continuous sutures (4/0 prolene). The drain was removed after the animals restored spontaneous breathing, followed by extubation.

### Experimental design

Three sets of experiments were performed.

First, to establish the safety of *in vivo* electroporation as method of gene transfer to the beating heart. The animals underwent gene transfer as described, with different pulsing parameters of 8, 16 and 32 pulses. Animals were evaluated for different parameters of toxicity at different time points.

Second, to establish *in vivo* electroporation as a method of gene transfer to the beating heart. Two groups were studied (n = 5). All animals underwent gene transfer as described. In the control group, the entire procedure was identical with the exception that the animals were not exposed to the electric pulses. Firefly luciferase under control of CMV immediate early promoter/enhancer was used and the animals were sacrificed 1 day after gene transfer. One animal was injected with 150 µl EGFP expression plasmid and electroporated as described above to localize the gene expression.

Third, to evaluate transgene expression over time. All animals underwent gene transfer as described and subgroups were sacrificed at day 1, 3, 5 and 7 after gene transfer (n = 4 per subgroup).As reporter gene firefly luciferase under control of CMV immediate early promoter/enhancer was used.

### Analysis of Toxicity

#### Haemodynamic Measurements

Rats were anaesthetized with isoflurane. The right carotid artery was exposed and a 1.4-French pressure micro-catheter (Millar®, Mikro-Tip®, Millar Instruments, Houston, TX, USA) was inserted into the left ventricle (LV). LV pressure curves were recorded and analyzed (PowerLab system and its Chart 5.2 software, AD Instruments, Spechbach, Germany). Data were recorded before the electroporation procedure (basal), then continuously over a 60 minutes period. Thereafter, in a subgroup of animals, (n = 4) data were measured 6 hours following the electroporation.

#### Cardiac Enzyme measurements

Two early cardiac specific biomarkers, CK-MB (Creatine kinase) and TnT (Troponin T) were used as indicators of myocardial injury. Blood samples were collected 6 hours following the electroporation, centrifuged and the serum was frozen at −20°C. Serum TnT and CK-MB were measured using commercial kits (respectively Roche Diagnostics, Basel Switzerland and Meia, Abbott, Baar, Switzerland) according to manufacturer's instructions.

#### Histological analysis at each time point

Immediately following euthanasia, the heart was placed in a container with 4% formalin for 12 hrs. After paraffin embedding the sections where cut and routine haematoxylin and eosin staining was performed. The sections were blinded and reviewed by an experienced pathologist. Gene expression was analyzed at day 1, 3, 5, and 7 following the *in vivo* electroporation procedure.

#### Bioluminescent reporter imaging (BLI)

The animals were anesthetized with thiopental (50 mg/kg Penthotal, Abbot AG, Baar, Switzerland). Monosodium luciferin (750 µl of an 80 mg/ml solution, Molecular Probes, The Netherlands) was injected i.p, 15 min before the heart was removed and imaged in an intensified charge-coupled device (CCD) camera (C2400-32, Hamamatsu), fitted with a 50 mm Nikkor lens (Nikon, Japan) and an image processor (Argus 20, Hamamatsu). An imaging system similar to that described by Contag et al [18] was employed for these studies as previously described [19].

#### Quantification of the Bioluminescent Signal

The luciferase activity was measured by counting the photon emission in the defined region of interest (ROI) with the aid of the open lab software (Improvision, Coventry, UK) [19].

#### Measurement of reporter gene expression

Slices of the hearts were frozen in liquid nitrogen immediately after imaging. The tissue was grinded and suspended in 1 ml of lysis buffer (Promega, Madison, WI, USA) and homogenized immediately. Samples were thawed at room temperature and vortexed for 30 sec. Subsequently they were refrozen in liquid nitrogen. Three freeze-thaw cycles were performed by alternating between liquid nitrogen and room temperature water bath. Debris was removed by centrifugation. Luciferase activity was then measured and expressed as RLU/mg protein, using the slow glow assay system (Promega, Madison WI) in MiniLumat LB 9506 luminometer (Berthold Technologies, Switzerland).

#### EGFP reporter gene expression and confocal microscopy

At day one after electroporation mediated gene transfer, the animal was sacrificed and the heart was fixed in O.C.T compound, the cryosections were made at the 10 µm thickness and sections were observed under LSM 510 exciter confocal microscope from Carl Zeiss (Germany).

#### Statistics

Data are presented as mean±SEM. The non parametric Mann Whitney U test was performed to compare the groups using GraphPad Prism version 4.00 for Windows, GraphPad Prism version 4.00 for Windows, GraphPad Software, San Diego California USA, www.graphpad.com. p<0.05 was taken as the level of significance.

## Results

### Electroporation mediated gene transfer to the beating heart is safe

Electroporation mediated gene transfer to the beating heart is safe Fifty-eight animals were used in the entire study. Overall mortality was 3.4% (2/58), due to bleeding from the coronary vein. Therefore, mortality was never related to the electroporation procedure itself. Especially, mortality remained null among the two groups of animals treated with the higher electroporation doses of 16 and 32 pulses.

Nevertheless, a very transient asystole followed the electroporation in 2/8 animals in the 32 pulses protocol. The asystole resumed spontaneously within 5 seconds. No fibrillation or other rhythm alteration was recorded.

**Figure 2:**
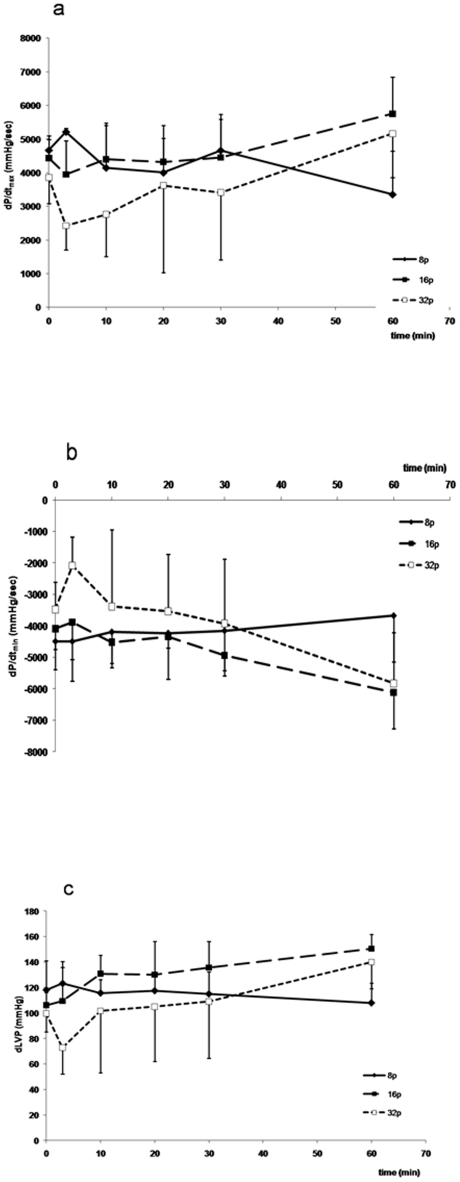
Left ventricular contractile function of heart following electroporation mediated gene transfer using 8, 16 and 32 pulses protocols (n = 5 each group). Indices of contractility and relaxation determined respectively by the maximum (dP/dt max) (a) and the minimum (dP/dt min) (b) and developed LVP (dLVP) (c) of the first derivative of ventricular pressure with respect to time. These parameters remained unchanged before (time 0) and after electroporation except for the 32 pulses group where the values are transiently altered. A complete normalization of the values occurred within 10–20 minutes. No abnormality was detected in the group in which invivo electroporation was not performed.

LV contractility function was analyzed over the first hour following the electroporation procedure. Contractility (LVdp/dt max) and relaxation (LVdP/dt min) as well as the developed LVP (dLVP) were altered after the electroporation procedure only in the group of animals with the maximum pulses regimen of 32 pulses (Figure 2); However, these functional alterations were rapidly reversible and complete normalization occurred within 10–20 minutes and persisted thereafter. Conversely, contractile function remained within a normal range in animals' electroporated with either 8 or 16 pulses.

**Figure 3:**
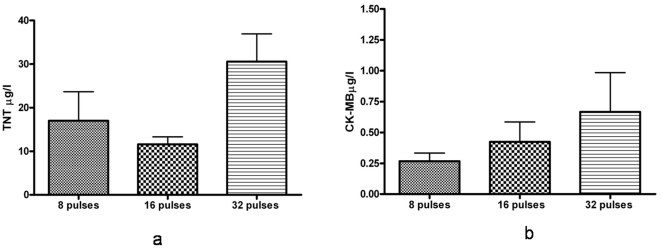
The two early cardiac enzymes for injury TnT and CK-MB were measured; both enzymes remained within normal range after *in vivo* electroporation mediated gene transfer with 8 and 16 pulses, but the values were altered with the 32 pulses protocol. (a) TnT levels (b) CK-MB levels. The levels were below detection levels in the group in which invivo electroporation was not performed.

**Figure 4:**
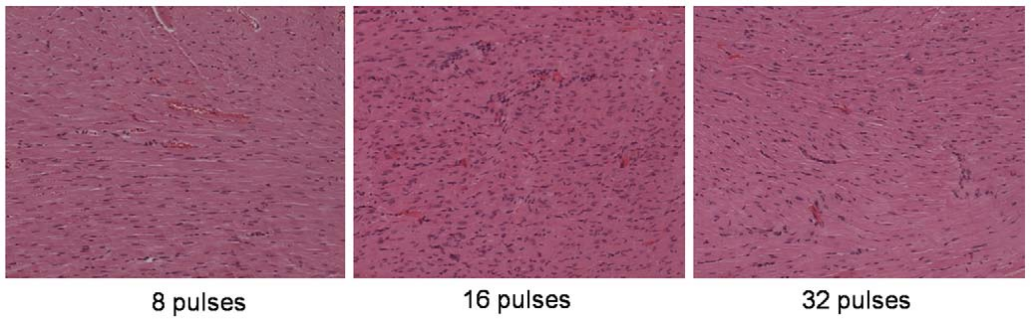
For histological analysis the hearts were evaluated at 24 hrs post gene transfer. The following criteria were considered: vascular congestion, infiltration and polymorphonuclear infiltrates. No haemorrhage or infiltration was noticed with any pulsing protocol. (a) 8 pulses (b) 16 pulses (c) 32 pulses (Magnification = ×200).

Six hours post-electroporation TNT (Figure 3a) and CK-MB (Figure 3b) values also remained in the normal range in animals treated with 8 and 16 pulses; Values were altered only when a 32 pulses regimen was tested (30.6±6.3 µg/l for TnT, and 0.66±0.318 µg/l for CK-MB). The levels in the control group without electroporation, were below detection value. Histological analysis performed at each time point, revealed no signs of fibrosis or necrosis however very moderate blood stasis, and very few interstitial lymphocytes, were observed after 8 pulses. For 16 pulses and 32 pulses slightly more blood stasis was observed with some interstitial and perivascular lymphocytes, but no signs of necrosis or fibrosis was seen (Figure 4).

### *In vivo* electroporation mediated gene transfer to the heart increases gene expression

**Figure 5:**
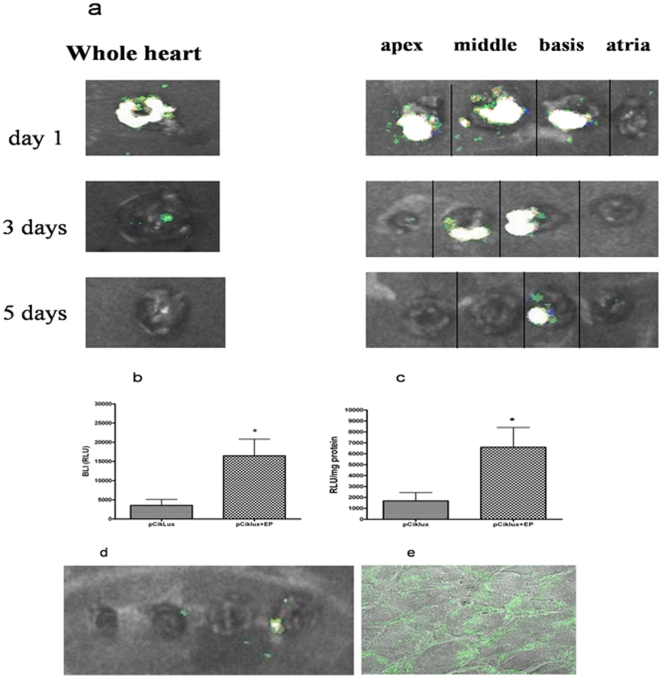
*In vivo* electroporation mediated gene transfer to the beating heart. (a) BLI images at day 1, 3 and 5 after *in vivo* electroporation mediated gene transfer to the normal heart. Graphical representation of the quantification of the luciferase activity, with electroporated compared to non-electroporated hearts; (b) BLI measurements, the photon counts per heart field are expressed in relative light units (RLU) 16479±4338 RLU for electroporated heart vs. 3539±1555 RLU for non electroporated heart (p = 0.018) at day 1. (c) Luciferase assay, RLU per milligram (mg) of protein was measured using a luminometer in the same sample 6594±1806 RLU/mg for electroporated heart vs 1684±760 RLU/mg of total protein for non electroporated heart (p = 0.011) at day 1. (d) Control heart, plasmid was injected but not elecroporated. (e) Expression of EGFP was found on the cell surface.

**Figure 6:**
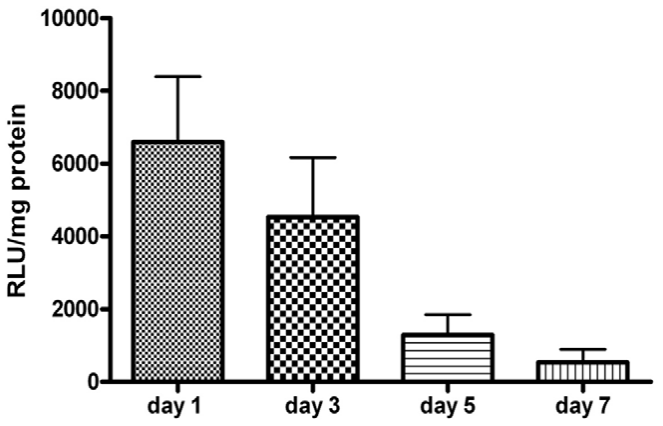
Graphical representation of transgene expression measured over time. After electroporation mediated gene transfer the animals were sacrificed at different time points.

When 150 µl of plasmid DNA pCiklux was injected without electroporation very low transgene expression was detected by BLI imaging (Figure 5 d). In contrast, when field strength of 200 V/cm and 8 pulses was applied after injection of 150 µg of plasmid pCiklux the mean lux activity was easily detected with BLI imaging at day 1 (Figure 5a). The luciferase activity was measured by counting the photon emission in the defined region of interest (ROI), RLU measured were 16479±4338 RLU for electroporated heart vs. 3539±1555 RLU for non electroporated heart (p = 0.018) (Figure 5b). These findings were further confirmed with conventional luciferase assay in the same samples. Luciferase activity observed on day 1 after electroporation mediated gene transfer was highest; 6594±1806 RLU/mg for electroporated heart vs 1684±760 RLU/mg of total protein for non electroporated heart (p = 0.011) (Figure 5c); however, the activity showed a gradual decrease, and at day 7 no transgene expression was seen (Figure 6). The confocal images showed the expression of the EGFP on the cell surface after *invivo* electroporation mediated gene transfer of the EGFP plasmid (Figure 5 e).

## Discussion

Regenerative medicine is a rapidly evolving field and efficient and reliable methods are needed to expedite the process of regeneration and healing. Current study demonstrates that *in vivo* electroporation mediated gene transfer to the beating heart is a feasible novel approach; it is also safe, quick, efficient and reproducible. After retrograde injection of plasmid DNA into the transiently occluded coronary sinus and immediate local application of a series of 8 electric pulses, a high gene expression can be achieved with no obvious adverse effect.

With the shortcomings of viral vectors and the inefficiency of the other non-viral vectors, the physical method of electroporation mediated gene transfer has emerged as a promising tool. Recently the use of this technique *in vivo* has gained increasing acceptance and has been used successfully in many tissues and organs [8], [11], [20], under various conditions [12], [14], [21], [22]. Even though interest has been shown towards this promising approach, yet its use in the cardiac muscles is limited. Few prior investigations reported the use of electroporation mediated gene transfer to cardiac tissue ex-vivo. Harrisson *et al* [15] electroporated chick embryonic hearts *ex vivo* by placing the heart in a bath containing the plasmid DNA, and showed high GFP and luciferase expression after 48 hrs. Similarly in the mice heterotopic heart transplant model Wang *et al* [16] demonstrated that *ex vivo* electroporation mediated gene transfer of the graft before its implantation, allowed significant gene expression. Very recently this technique was also applied on the pig heart [23]. Therefore, it was with no doubt that electroporation would enhance gene expression in the cardiac muscle. In the current study, however we demonstrate the applicability of this technique with ease and safety on beating hearts of normal rats.

Various routes of administration of the plasmids in the heart have been reported, but none of the protocols could achieve satisfactory global distribution in the heart, especially not in animals with heart failure. Some studies have evaluated the easy direct injection of plasmid into the heart muscles as an efficient method. It is however limited by local needle injury and time consuming electromechanical mapping techniques [24]. The epicardial delivery is another approach where the natural cavity formed by the pericardial pouch was thought to be an advantage. The higher permeability of the pericardium as compared to the epicardium, lets the injected solution diffuse more eccentrically than concentrically [25]. Most recently the percutaneous transluminal approach gathered a lot of attention as specially designed ventricular catheters allow direct injection within the myocardium [26], however, the problem of local needle injury and adequate distribution still remains. In laboratory experiments the coronary delivery approach has been largely used by cross clamping of the ascending aorta and immediate injection of the gene solution to the left ventricular cavity, the solution being delivered to perfuse both coronaries [27]. Unfortunately limitations of this technique involve an acute elevation of the afterload that will further reduce LV function following aortic cross clamping. In the present study we evaluated a retrograde approach where the coronary sinus is clamped transiently before the plasmid solution is injected retrograde into the coronary veins. Using this approach, we were able to achieve global transgene expression in the heart, hence opening new ways of delivering genes to failing hearts.

Since application of direct defibrillation to the heart is almost a routine procedure at the end of cardiac surgery procedures, hence *in vivo* electroporation mediated gene transfer to the beating heart could easily be applied intraoperatively in routine clinical practice. Numerous studies of myocardial damage caused by electrical currents have been performed [28] and experiments have been conducted to evaluate the toxic effect of electroporation mediated gene transfer to the myocardium [29], [30]. It has been shown recently that electroporation induces only very transient phenotypic and morphological alterations of skeletal muscle fibres [31]. In our current study, we also systematically analyzed the possible side effects of *in vivo* electroporation on the cardiac muscle including biochemical, functional and histological parameters. In the group treated with 8 and 16 pulses, no alteration in LV function was observed; transient asystole and reduced contractile function as well as slightly elevated levels of TNT and CK-MB were observed only when 32 pulses were administered. Histological assessment revealed no signs of injury, haemorrhage or infiltration.

Although the results are very promising, the technique has a few limitations. Electroporation is a novel approach and needs practice; indeed, the injection has to be performed very diligently since the coronary vein is fragile. There may be concern about the homogenous distribution with retrograde injection, as compared to the antegrade approach. However, the retrograde approach offers the major advantage that it can perfuse ischemic areas whereas the antegrade delivery is limited by coronary stenosis and/or occlusion. For effective gene therapy of chronic disease, persistent transgene expression is needed. In this present study we have used the CMV early promoter enhancer which allows early and transient expression; undergoing transcriptional inactivation [32], [33]. Further studies are needed with more advanced promoter systems which could provide prolonged transgene expression [13] and also this technique should be tested in infracted heart.

In conclusion, this initial study demonstrates that *in vivo* electroporation mediated gene transfer to the beating heart is feasible and safe. A high gene expression can be achieved in normal hearts. These observations make *in vivo* electroporation an attractive alternative to commonly used delivery techniques for gene therapy for heart disease. However, further evaluations including the use of functional genes are required to further evaluate this technique before clinical applications.
